# Designing and Governing Responsive Local Care Systems – Insights from a Scoping Review of Paramedics in Integrated Models of Care

**DOI:** 10.5334/ijic.6418

**Published:** 2022-04-13

**Authors:** Amir Allana, Walter Tavares, Andrew D. Pinto, Kerry Kuluski

**Affiliations:** 1 Institute of Health Policy, Management and Evaluation, Dalla Lana School of Public Health, University of Toronto, CA; 2 McNally Project for Paramedicine Research, CA; 3 Upstream Lab, MAP/Centre for Urban Health Solutions, Li Ka Shing Knowledge Institute, Unity Health Toronto, CA; 4 The Wilson Centre and Temerty Faculty of Medicine, University of Toronto|University Health Network, CA; 5 York Region Paramedic Services, Community and Health Services Department, The Regional Municipality of York, CA; 6 Department of Family and Community Medicine, Faculty of Medicine, University of Toronto, CA; 7 Institute for Better Health, Trillium Health Partners, CA

**Keywords:** integration, paramedic, local, adaptability, flexibility, workforce, governance

## Abstract

**Introduction:**

Programs that fill gaps in fractured health and social services in response to local needs can provide insight on enacting integrated care. Grassroots programs and the changing roles of paramedics within them were analyzed to explore how the health workforce, organizations and governance could support integrated care.

**Methods:**

A study was conducted following Arksey and O’Malley’s method for scoping reviews, using Valentijn’s Rainbow Model of Integrated Care as an organizing framework. Qualitative content analysis was done on clinical, professional, organizational, system, functional and normative aspects of integration. Common patterns, challenges and gaps were documented.

**Results:**

After literature search and screening, 137 documents with 108 unique programs were analysed. Paramedics bridge reactive and preventative care for a spectrum of population needs through partnerships with hospitals, social services, primary care and public health. Programs encountered challenges with role delineation, segregated organizations, regulation and tensions in professional norms.

**Discussion:**

Five concepts were identified for fostering integrated care in local systems: single point-of-entry care pathways; flexible and mobile workforce; geographically-based cross-cutting organizations; permissive regulation; and assessing system-level value.

**Conclusion:**

Integrated care may be supported by a generalist health workforce, through cross-cutting organizations that work across silos, and legislation that balances standardization with flexibility.

## Introduction

Despite decades of policy efforts by governments to integrate care [1,2,3], fractured and disjointed systems persist, resulting in gaps in services. Integrated care can be defined as when “network[s] of multiple professionals and organisations across the health and social care system provide accessible, comprehensive and coordinated services to a population in a community” [4 p. 3]. However, health systems continue to face challenges bridging episodic, acute care with individualized chronic and continuing care [5]; health and social services are often siloed [6]; and inadequate primary and preventative care can result in higher acute care utilization [7,8]. These challenges prevent communities from realizing the improvements – in client experience, value for money, disease prevention and population health – that integrated care purports to offer [9,10].

It has been suggested that attempts at integration fail in part due to bureaucratic, command-and-control approaches to management and policy, rather than fostering an enabling environment for collaboration to emerge more organically between professions and organizations [11,12]. Two areas of research for fostering an enabling environment for integrated care have been around evolution of the health workforce [13,14] and collaborative governance [15]. Changes to the health workforce include the need for flexible, agile staffing models, multidisciplinary practice, reducing siloes between professions and guidance on skill-mix for different care teams [13,14,16]. Collaborative governance refers to mechanisms for integrated care teams and organizations to take joint accountability and action on shared goals [15]. While integrated care frameworks have long maintained that coordinated service delivery relies on integration at multiple levels [17,18], research is still evolving on how to do this in a way that enables integration from the ground-up rather than enforcing it from the top-down.

Examining attempts at integrated care, particularly instances of ‘ground-up’ initiatives, can provide insights into how health systems can address key gaps in services and better enable and support integration. Instances of ‘ground-up’ local innovation – i.e., programs developed on a small-scale and not part of system-wide plans or designs – often emerge in settings where fractured and disconnected care serve as an impetus for grassroots initiatives to fill gaps in services. These initiatives can emerge even in the absence of supporting professional, organizational, governance and policy mechanisms [19]. For instance, over the past two decades, local innovation has driven an expansion in the paramedic profession [20,21] beyond it’s traditional role of emergency ambulance transport [22]. Paramedics are now part of palliative care teams [23], they conduct community paramedicine home visits [24] and provide on-site preventative care in congregate living settings [25]. Despite being credited as examples of patient-centred, integrated care [26], these localized adaptations in paramedics’ roles have resulted in tensions around professional role definitions [27,28], regulatory and legal frameworks [29,30] and funding models [31]. This suggests that the professional, organizational and system-level environments are not conducive to these programs, and may explain why initiatives involving paramedicine face challenges with scope and sustainability [32,33,34].

Grassroots programs in which paramedics are involved appear to be bridging gaps between acute and chronic, hospital- and community-based care; analyzing them can elucidate how to systematically address these care integration challenges. While several reviews have described the clinical functions and educational needs of newer paramedic designations such as ‘community paramedicine,’ [20,35] no previous studies have examined paramedics in the context of integrated care. The role of paramedics in integrated care teams is understudied and provides a setting in which to explore questions in the field of integrated care such as: types of service gaps local innovations attempt to fill, characteristics of an integrated care workforce, responsive organizational models and enabling legislation.

This scoping review was conducted to address the overarching research question: what can be learned about designing and implementing integrated care from the literature on programs and initiatives in which paramedics are involved? This involved two sub-questions:

– What populations and gaps in care are being addressed by paramedics?
– What do the experiences of grassroots programs suggest for governing health systems towards improved collaboration and responsiveness to local population needs?

The study objectives were to:

– describe key features and approaches to integration in programs where paramedics are involved;
– develop a conceptual synthesis based on common patterns, challenges and knowledge gaps in the data; and
– discuss the implications for integrated care broadly, including service design, workforce skill mix and system governance.

Through pursuing these objectives, this study identifies key insights that can be useful to integrated care experts, policy makers, health system leaders and the paramedic community. By examining how grassroots attempts at care integration operate and structure themselves – and the challenges encountered – this study adds to the integrated care discourse on workforce skill mix and system governance. The results of this study may also help the paramedic profession contextualize their role within integrated care models.

## Methods

This study followed the revised version of Arksey and O’Malley’s scoping review methodology [36] as described by Levac et al. [37], following reporting guidelines from the Joanna Briggs Institute [38] and the PRISMA extension for scoping reviews (PRISMA-ScR) [39]. The study protocol was publicly registered prior to commencing and remains available for review [40]. Valentijn’s Rainbow Model of Integrated Care (RMIC) taxonomy [41] was used as an organizing framework. The RMIC was chosen over other frameworks as it offered an open-ended, exploratory, broad and comprehensive view of integrated care that could be applied to various, diverse models of care. Key RMIC concepts, summarized in ***Table 1***, were used in developing the literature search, data collection and analysis methods. This ensured that consideration was given to different types of integration (clinical, professional, organizational, system, functional and normative) at multiple levels (micro, meso and macro) and to varying degrees (linkage, coordination, full integration). Findings were synthesized and interpreted with reference to the broader integrated care literature.

**Table 1:** Integrated care concepts and definitions, based on Valentijn’s Rainbow Model taxonomy, used to inform study design and analysis.


CONCEPT	DEFINITION (BASED ON VALENTIJN ET AL. [41])

Principles of Integration	Underlying program philosophy, how the target population is defined, and reasoning or purpose of the program or initiative.

Breadth of Integration (Vertical vs Horizontal)	Vertical integration is rooted in a disease-focussed paradigm where care is escalated from generalists to specialists. Horizontal integration is rooted in a primary care and public health paradigm with an emphasis on ongoing, holistic and preventative health services.

Degree (or extent) of Integration	Expressed as a scale from segregation (no integration), linkage (low-level integration, connections and referrals), coordination (medium-level integration, active coordination of professions and organizations) to full integration (team-based care with pooled resources and shared management).

Clinical Integration (Micro-level)	Case management and polices to identify clients with specific risk profiles, care processes that ensure continuity, interactions between the provider and the client and the use of individualized multidisciplinary care plans.

Professional Integration (Meso-level)	Interprofessional education with a focus on collaboration; service delivery agreements between providers; and value creation for the professional.

Organizational Integration (Meso-level)	Governance structures amongst the organizations involved. Mechanisms for joint accountability and policies, having an explicit organizational strategy and the degree of openness and trust between organizations.

System Integration (Macro-level)	Alignment of regulatory frameworks, market dynamics and political and social climate to support integrated care.

Functional Enablers	Learning infrastructure for joint research and development; aligned information management, information sharing and benchmarking; and regular feedback on performance to enable quality improvement.

Normative Enablers	Having a shared long-term vision, the extent to which agreements are fulfilled, how reliable and predictable the behaviour of different team members is, strong leadership that mobilizes towards a shared vision, and linking cultures and values within the model.


Levac outlines six stages for scoping studies [37]: (1) identifying the research question, purpose and intended outcome; (2) identifying relevant studies; (3) study selection; (4) charting the data; (5) collating, summarizing and reporting the results; and (6) consultation. The literature was searched broadly for all examples where paramedics interact with one or more other service provider. Data was charted using the RMIC concepts in ***Table 1*** and analyzed in three stages as per Levac’s suggestions, with some interpretation throughout [42]: qualitative content analysis; grouping of common concepts; and considering the meaning of findings in relation to the research questions. A synthesis of results was reported followed by a discussion on implications for integrated care. The study team’s experience and expertise in paramedicine and integrated care influenced how results were interpreted and discussed. Experts from the integrated care and paramedicine community were consulted to contextualize the findings.

### Identifying relevant studies

The literature was searched using a comprehensive, inclusive set of keywords related to *integrated care* (e.g., “care coordination”, “collaboration”, or “team-based care”) and *paramedicine* (e.g., “paramedic”, “emergency medical technician”, or “ambulance”). Additional keywords were included for novel designations of paramedic practice such as “paramedic practitioner” and “community paramedic”. The following databases were queried using MeSH subject headings and keywords: MEDLINE, Embase, CINAHL, PsychInfo, and Cochrane. All queries were run on March 20 2020. A sample search query is provided in Supplemental File 1.

Grey literature was searched using the OpenGrey database, the COS Conference Papers Index, and the first 200 results from Advanced Google. References of relevant literature reviews [20,26,43] were checked to identify additional studies. Where database searching revealed multiple relevant studies from certain universities, their institutional repositories were searched for dissertations. These universities were: McMaster, University of Toronto, Dalhousie, Monash, and Swansea. Grey literature search was conducted between 1–4 January 2021. All search terms and results were documented to preserve an audit trail.

### Study selection

Inclusion and exclusion criteria were developed to be broadly inclusive (e.g., including referral pathways between providers and organizations) while excluding studies that only mentioned the traditional transport function of paramedics. Given the variation in paramedicine around the world, only programs based in nine OECD peer countries [44] with comparable health systems were included. For the purposes of this study, “paramedics” were defined as a unique profession distinct from other health professionals such as nurses or physicians, working in a plurality of settings including ambulances, clinics and hospitals [45]. In addition to any job title that included the word “paramedic,” consideration was given to international differences by including commonly used job classifiers such as “emergency medical technician” and “ambulance clinician” (see Supplemental File 1). Given the recent, significant restructuring and professionalization of paramedics around the world, only literature from the past two decades was included so that findings were relevant to present-day health systems.

Documents were included if they met all of the following criteria:

– care process or program was described where paramedics and at least one other type of care provider were involved, including consultation or referral;
– paramedics were described to be playing a role beyond indiscriminately transporting patients to an emergency department, including any decision-making regarding treatment or transport;
– based in the following countries: Canada, Australia, France, Germany, Netherlands, New Zealand, Sweden, the United Kingdom or the United States;
– available in English; and
– published in 2001 onwards.

Documents were excluded if they met any of the following criteria:

– did not describe a specific program that had been implemented;
– study protocols, literature reviews and policy statements;
– brochures, newsletters and slide decks; or
– had no accompanying full-text publication (i.e., conference abstract only).

Following Levac’s suggestions, selection criteria were developed iteratively and reviewers met multiple times during the screening process to discuss uncertainties. Inclusion and exclusion criteria were pilot tested by two reviewers on the titles and abstracts of 200 randomly-chosen citations. Criteria were revised for clarity iteratively until the reviewers were consistently in agreement (inter-rater agreement of >80%) [46]. Two reviewers split up the remaining citations for title and abstract screening. During full-text screening, each citation was reviewed by two reviewers and all conflicts were discussed and resolved through consensus.

### Charting the data

A data extraction form was built collectively by the study team based on the RMIC taxonomy (***Table 1***). Data extraction prompts were written to be open-ended and broad to minimize imposing the study team’s preconceptions on the data. The form was pilot tested on a set of five citations and the entire study team met to discuss and revise the form. One member completed all data extraction, writing memos along the way regarding emerging patterns and concepts, and regularly reporting back to the study team. Throughout the data charting process, document authors’ own language was used –with direct quotes and some paraphrasing – and interpretation was minimized.

### Collating, summarizing and reporting results

Analysis was done in three stages: (1) descriptive qualitative content analysis [47,48,49]; (2) synthesis of common concepts and ideas; (3) interpretation of results in relation to the research questions. First, a full reading of the charted data was done to garner a general impression. Based on this general impression, memos from data extraction and the RMIC taxonomy, categories were developed within which the data was coded deductively. Categories included RMIC concepts such as: target population, program rationale, clinical features, professional features, organizational relationships, system-level policy and regulation, functional enablers and information sharing, normative enablers and culture. Coding was done iteratively in batches of 10–12 citations and a codebook was maintained. Codes were developed based on similar words and phrases used by document authors and refined throughout the coding process. As coding proceeded, some codes were merged while others were discarded (for reasons such as lack of adequate data and redundancy). Results were aggregated and organized using the RMIC taxonomy. Programs with similar characteristics were clustered together to develop a synthesis. Results are reported in three parts: (1) numerical summary of study characteristics; (2) table of descriptive findings from qualitative content analysis; (3) synthesis. Finally, the study team met to discuss the synthesized results and interpreted them in relation to current discourse in integrated care, including workforce skill mix, governance and regulation. The team discerned key cross-cutting concepts that would be relevant for integrated care researchers and health system leaders. This led to the development of the five concepts described in the ‘discussion’ section of this paper.

## Results

**Figure 1:**
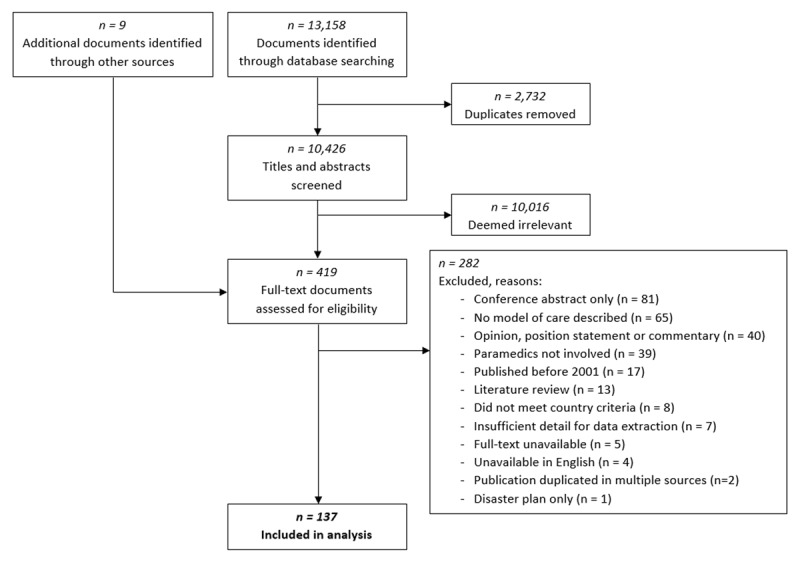
PRISMA flow diagram for document searching, screening and inclusion.

After database searching and removal of duplicates, 10,426 unique citations were identified. Nine additional documents were found during the grey literature search. As detailed in the flow diagram (***Figure 1***), after screening for inclusion and exclusion criteria, 137 documents were included in the analysis. Among the 137 documents, at least 108 different programs or initiatives were identified. A complete list of documents and description of programs are provided in Supplemental File 2.

The 137 documents included in the analysis were from 71 different journals or publications. A majority of the literature was comprised of observational studies (n = 44, 32%), vignettes (n = 43, 31%) and case reports (n = 22, 16%). Peer-reviewed journals constituted 72% of the literature (n = 98); the remainder were non-peer-reviewed magazines and trade journals. Over 80% of the literature originated from the United States, Canada and the United Kingdom; 45% were based exclusively in urban or suburban settings and 18% in rural and remote areas. Where the start date of the program or initiative was provided, a majority began implementation between 2006 and 2015 (n = 68, 69%, excluding missing data).

Descriptive results from qualitative content analysis are presented in ***Table 2***, with additional detail in Supplemental File 2. There was a higher volume of information on clinical and professional aspects of integration – such as services provided (n = 119, 87%) and providers involved (n = 122, 89%) – than organizational and system-level aspects such as governance models or regulations (n = 57, 42%). There was least information on the normative enablers of integration such as team culture and values (n = 39, 28%). Programs and initiatives with similar target populations and care processes were grouped together and examined for common features and gaps in integration. A qualitative synthesis of these findings is presented in the following sections.

**Table 2:** Descriptive summary from qualitative content analysis with example citations.


CATEGORY	SUMMARY OF FINDINGS AND EXAMPLE CITATIONS

Target populations	– People at high risk for hospital readmission [50,51,52,53] – High utilizers of emergency services [54,55,56] – Emergency episodes: mental health [57,58,59,60], heart attacks [61,62,63,64,65], strokes [66,67,68], low-acuity injuries [69,70,71,72,73]; falls [74,75,76,77]; and hypoglycemia [78,79,80] – People with multiple chronic diseases such as congestive heart failure (CHF) and chronic obstructive pulmonary disease (COPD) [81,82,83] – People with complex needs: palliative care [84], long-term care [85,86,87,88] – Older adults and seniors living in the community [89,90,91,92,93]

Rationale, drivers or goals of program	– Reduce unnecessary use of hospital and emergency services [52,90,94,95] – Improve patient experience and appropriateness of care [54,69,84,86,96] – Respond to cost, staffing and resource pressures [97,98,99,100] – Improve care access in isolated rural areas with limited staff [101,102,103,104,105] – Improve health promotion and prevention [106,107] – Improve patient health outcomes, decrease morbidity and mortality [61,63,66,108,109] – Improve access to care for hard-to-reach populations such as people who are homeless and undocumented [55]

Clinical features (Micro-level)	– Unscheduled, on-demand episodic care accessed via: universal emergency phone number such as 911, 999 or 111 [55,61,62,69,74,79,96,97,110,111] dedicated non-emergency number for enrolled populations [83,112,113,114] – Scheduled and drop-in services: in clients’ homes [51,72,102,108,115,116], community spaces [90,111,117,118] or mobile pop-up clinics [119] – Community-based follow-up care: social work [55,120,121,122], home care nursing [56,121], diabetes clinics [79,80], falls prevention teams [75,77] and mental health facilities [57,59,123,124,125] – Clients with certain risk profiles rostered to programs by primary care providers [81,82,92,112,126], hospitals [51,52,95,108,120,127], or by paramedic services [55,56,128] – Case management and coordination for clients with multiple needs [52,54,129,130]

Professional features (Meso-level)	– Informal, ad-hoc collaboration and consultation with primary care physicians, community nurses, pharmacists and social workers [50,55,69,83,90] – Formal, protocol-based collaboration with specialists: cardiology [62,64,109,131,132,133], neurology [66] and psychiatry and mental health [110] – Mobile teams of paramedics, pharmacists, nurses and social workers [55,57,72,116,120] – Critical care teams consisting of paramedics, nurses and physicians providing specialized care [134,135,136,137,138] – Paramedics as autonomous practitioners with independent decision-making [96,97,125,139] – Paramedics as “physician extenders” who implement physician orders [69,81,140,141] – Role confusion and interprofessional tensions [117,141,142,143]

Organizational relationships (Meso-level)	– Service-delivery partnerships: agreement between a paramedic service and a hospital for provision of post-discharge home visits [51,94,108,127] agreement between a paramedic service and a primary care team to respond to acute exacerbation of symptoms for their clients [81,112] – Formal taskforce, committee or coordinating entity with joint accountability and shared decision-making [62,91,105,114,144] – Being geographically dispersed in a catchment area working across traditional jurisdictional boundaries [83,84,120,141] – Sharing of paramedic staff with other agencies: general practices and urgent care [71,142], rural emergency departments [118,145]

System-level: policy, regulation and market dynamics (Macro-level)	– Need to assess value and cost-effectiveness at a system-level, misaligned reimbursement models [95,98,100,146] – Major policy drivers influence the development of new programs or initiatives: multi-professional working in the UK [72,114,147,148], financial penalties for 30-day readmissions in the USA [52,120,149,150] – Limitations of medical oversight mechanisms and medicolegal regulations [119,151,152] – Barriers from legal requirements that mandate transport to a hospital [69,110,115,140,146]

Functional enablers: information flows, data and benchmarks	– Benchmarks and measures of success: service utilization (e.g., number of patients, interventions performed) [73,76,97,134] time-based indicators (e.g., ambulance response time, total care duration, time to treatment) [64,66,131,132,137,138] measures of safety (e.g., rate of adverse events) [87,89,125,134] service avoidance (e.g., number of ED visits mitigated, length of hospital stays, readmissions) [57,76,82,85,89,90] patient satisfaction surveys [89,93,144,153] – Databases or patient registries for longitudinal studies [61,94,132,136,154] – Mechanisms to regularly re-evaluate, change or adapt the program in response to new insights [62,67,141,155] – Learnings from critical cases and feedback provided to staff [109,112,114,156] – Siloed, inadequate data or IT infrastructure as a limitation to evaluation; datasets managed by different organizations and not interoperable [55,69,111,121] – Information flow between paramedics and other providers: phone call, paper and fax [51,77,82,86,108,121]; one-way electronic transmission of referrals [79,91,157] – Real-time shared patient records between paramedics and other providers [83,112,114,128,158]

Normative enablers: culture and shared behaviours of the care team	– Tensions in norms around pace of care: faster, structured pace of emergency care versus the slower, uncertain pace of primary care [51,88,94,158,159] – Reconceptualizing relationship to risk: from risk avoidance to risk tolerance [141], damaging “domino effect” of activating emergency services leading to over-treatment and poor client experience [82,129] – Tensions between independent, autonomous paramedic practice and joint accountability in a care team [118,148]


### Models of care and degree of integration

**Figure 2:**
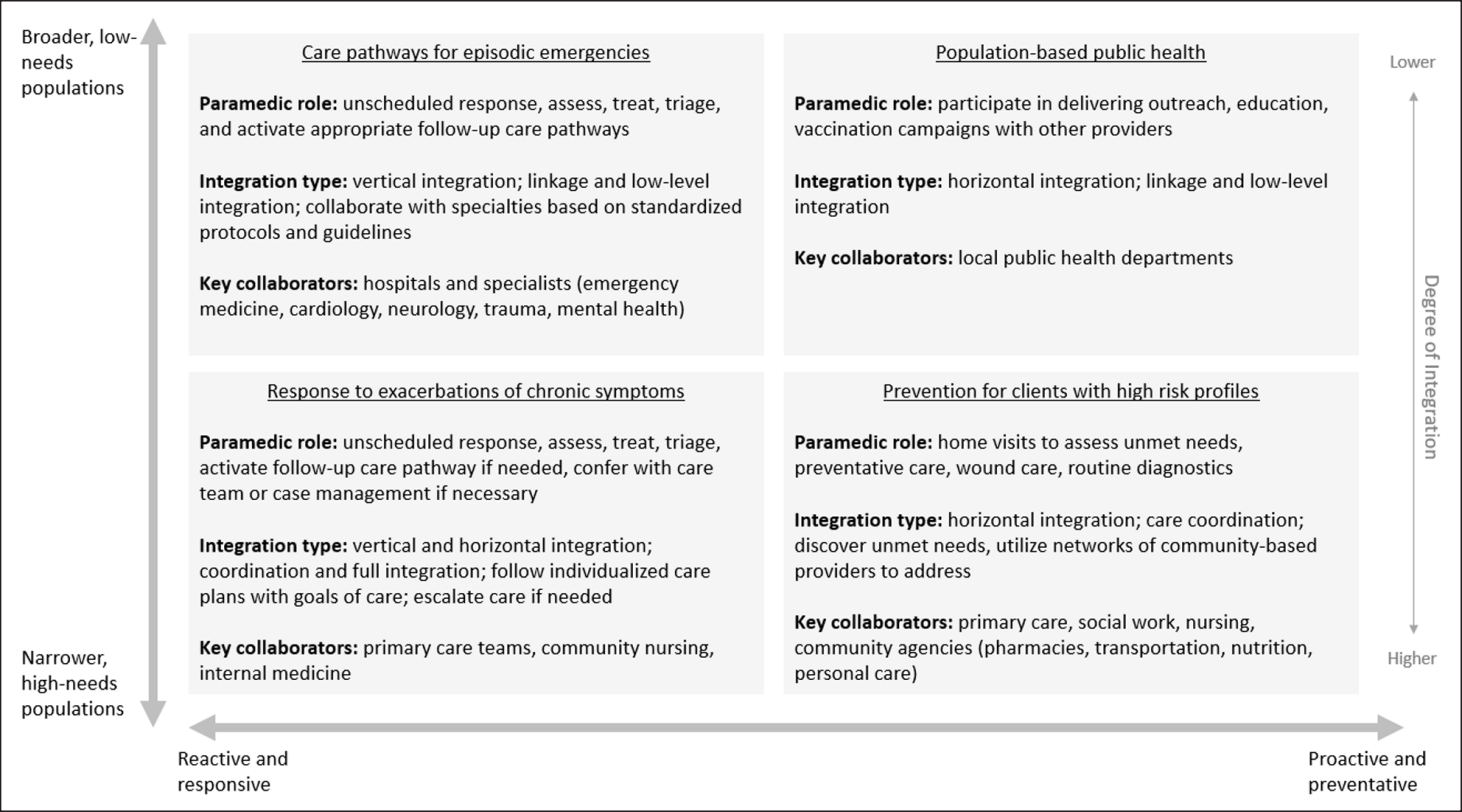
Types of integration and paramedic roles in reactive and proactive models of care for low- and high-needs populations.

By grouping programs and initiatives with similar features, the target populations, types of integration activities and potential gaps in services filled by paramedics were conceptualized in a grid, seen in ***Figure 2***. The grid shows two scales: reactive to preventative care, and complexity of population need. This divides the models of care in the literature into four domains; these are discussed below, starting with reactive services (left side of the grid) followed by preventative (right side of the grid).

The top-left of ***Figure 2*** describes episodic, reactive models of care for broad, low-needs populations experiencing an emergency event. This involves low-level linkage and vertical integration, where standardized clinical guidelines are used to refer or escalate care to the appropriate specialty. This expands on the traditional paramedic role to include activation of more specific care pathways for a client’s needs with the goal of avoiding emergency department use where possible. There are specific pathways for a growing number of episodic emergencies in the broad population, including heart attacks [61,62,63,64,65], strokes [66,67,68], mental health and addictions crises [57,58,59,60], falls in older adults [74,75,76,77], hypoglycemic events [78,79,80] and musculoskeletal injuries [69,70,71,72,73]. In addition to treatment and care navigation, paramedics play a case-finding role by identifying patients with certain risk profiles and initiating follow-up preventative care. For example, a hypoglycemic episode could suggest need for reassessment of a client’s diabetes management strategy; a fall could suggest a client needs an in-home falls risk assessment. These reassessments and preventative pathways are then activated by paramedics through conferring with and referring to other providers.

On the bottom-left of the grid are models of care to provide episodic, reactive services to populations with more complex needs experiencing an exacerbation of chronic symptoms. This involves a higher degree of integration, where care is guided by individualized care plans and goals of care set up ahead of time by the client’s care team [81,84,88,155]. Care may be escalated to specialists if required, or findings communicated back to the appropriate care teams and community agencies for follow-up. This form of integration tends to involve regular communication with a care team, shared client records and an understanding of the client’s unique context. Populations served by these models of care include: people with multiple chronic conditions, chronic respiratory and heart disease, people requiring long-term care and people in palliative care.

On the top-right of the grid are public health and prevention services for entire populations, including health education and vaccination campaigns [143,159,160]. Paramedics here are part of a shared health care workforce, playing overlapping roles with other providers such as public health nursing. Paramedic involvement in this domain is often driven by health human resource needs, particularly in rural areas with staff shortages and spare paramedic capacity due to a lower emergency case load.

On the bottom-right of the grid are models of care to provide preventative and proactive services for populations with certain risk profiles. Paramedics play the function of risk and needs identification, with their roles often overlapping with community-based nursing, social work and personal care providers. In these models there is more horizontal integration and care coordination across networks of providers: involvement of case managers, discovering unmet health and social needs and arrangement of wrap-around care from the appropriate agencies. These models of care are often activated by primary care teams [105,126], hospitals at the time of discharge [95,127], and paramedic services themselves based on repeat emergency calls from the same client [55,56].

### Key issues, challenges and gaps in integration

Across all programs, there was evidence of clinical integration such as case-finding, care pathways for individual client needs and addressing biopsychosocial factors. Specific clinical pathways varied and depended on local context. Care was often available to clients on-demand and occurred in home and community settings. Case finding was both opportunistic and intentional – with mechanisms for paramedics to activate preventative care or reassessment during any client encounter.

Professional integration was incomplete in the programs, with variable team composition and unclear role boundaries, even across programs with similar target populations and activities [57,58,59,60,124]. While there were localized agreements on cross-provider consultation – particularly between paramedics and physicians for performing medical procedures – there was confusion in interprofessional understanding. Many programs required additional skills training for paramedics which were specific to the initiative and developed in-house or provided by partner agencies, for example, training in medication reconciliation [52,104,120], wound care [85,93,100,103,104] and phlebotomy [93,103]. There was an absence of formal interprofessional education. Some literature mentioned prioritizing job satisfaction and staff retention [161,162], but there was generally a lack of emphasis on creating value for the professional.

There was minimal evidence of organizational integration amongst the programs. Most initiatives depended on partnerships between local organizations with independent management structures, performance indicators and finances. These partnerships were typically between paramedic services and hospitals, primary care or community agencies to provide a specified set of services (see ***Table 2***). In most examples, paramedic services maintained organizational segregation from their partner agencies, and some suggested that this allowed them to work across silos [157,161].

System-level policies and regulations appeared to be incompatible with some forms of integration being practiced, such as regulations that limited medical scope and prevented collaboration between paramedics and non-hospital-based agencies. This was a challenge for programs that, for example, provided care to clients whose wishes did not involve hospital-based care or for whom hospital care was inappropriate [81,84]. Programs also reported that payment systems disincentivized paramedics from providing some services by failing to capture the value of preventative and community-based care while incentivizing transport to hospitals [115,159].

Functional integration varied in terms of clinical information sharing, and there was little evidence of specific care quality measures being utilized. Information sharing about client health history was constrained to models of care that target narrow populations with complex needs, where some used shared electronic medical records between paramedics and primary care teams [83,112]. There was minimal differentiation in quality indicators and benchmarks for different models of care; most tended to measure generic indicators of speed, service utilization and safety. However, a few programs did measure specific quality benchmarks for a target population, for example diabetes management [78,80] and cardiac disease [109].

There was some evidence of tensions between the norms of paramedicine and those of preventative and primary care teams, but there was a low volume of literature that discussed normative integration. Two specific tensions related to: (a) how providers conceptualized risk and (b) degree of provider autonomy. The literature discussed paramedic norms of risk aversion – rooted in emergency medicine – as being in tension with more risk-tolerant primary and community care settings, particularly when providing preventative or individualized services [141]. Secondly, the literature discussed the tension between autonomous practice and joint, team-based practice. In the traditional emergency setting providers tend to work alone, and some programs discussed challenges with paramedics moving towards a culture of joint accountability [118,148].

## Discussion

The programs and initiatives analyzed in this study are responding to local needs by filling gaps in health and social services [20]. This study does not assess whether or not these specific programs lead to better care quality, nor if paramedics are the most appropriate provider to play some of these roles. However, the results of this study – in terms of how local programs structure themselves and the challenges they encounter – provide insight for fostering responsiveness, adaptability and connectivity in integrated care systems. Some key concepts arising from study findings are:

– Clinical pathways with a single point-of-entry
– A mobile, flexible and generalist workforce
– Role of a cross-cutting service organization
– Need for permissive regulation
– Assessing value at a systems level

These concepts should not be seen as mutually exclusive, but rather as intersecting ideas that together could foster more collaboration and connectivity in local systems [11]. Each of these concepts are discussed in the following sections.

### Clinical pathways with a single point-of-entry

The concept of a single point-of-entry is a key success factor for integrated care, particularly in developing comprehensive service packages for clients with higher needs [17,163]. The programs analyzed in this study tended to use a single point-of-entry for a broader spectrum of low- and high-needs populations with an emphasis on care navigation and case-finding at every provider-client interaction. In many models of care analyzed in this study, a client could access multiple services and clinical pathways through a single provider. Clients accessed services on-demand, often 24-hours-a-day, in homes and community settings. Activities and clinical pathways were not pre-determined prior to provider-client interaction, but rather emerged based on clinical assessments and dialogue with the client, and the providers were able to activate relevant reactive and preventative services. These pathways were sometimes individualized (e.g., for enrolled populations with care plans) or standardized based on clinical guidelines (e.g., individuals experiencing an episodic event). This may signal a public desire for more streamlined access to multiple care pathways, and single point-of-entry system designs could be considered for a broader spectrum of population needs.

### A mobile, flexible and generalist workforce

This study aligns with the literature that integrated care calls for a workforce that can be deployed flexibly across multiple clinical settings [13,14,164]. Paramedics appear to be playing some functions of an adaptable workforce: they are mobile in the community and able to be deployed as-needed, at all hours, serving different functions for a range of populations. This mobility, availability and timely responsiveness may be the value that drives some local systems to utilize paramedics to fill gaps in services. Importantly, rather than the creation of sub-specialties or new role designations, it is the same provider playing a spectrum of clinical roles in different settings albeit with some additional skills training. This is an argument for taking a generalist approach to the foundational competencies of the health workforce such that professionals are equipped and willing to work across different settings, supplemented by easily accessible training for specific skills on an as-needed basis. As has been suggested by previous studies in integrated care, this supports de-emphasizing unique professional identities in favor of a more shared workforce culture across the health and social care system [165]. If functional flexibility is desirable, then some overlap is likely unavoidable. Tensions between professional groups [19,165] can be mitigated through shared learning, job shadowing and job rotation opportunities within the workplace. Cross-disciplinary career pathways could also be considered, such as the creation of the Emergency Care Practitioner role in the UK [147] which allows the career pathways of nurses and paramedics to converge. However, this risks once again creating new designations and may reduce the flexibility offered by a single-tier, generalist workforce.

### Role of a cross-cutting service organization

Assuming gaps in services are a feature of even the most well-designed systems, there may be a role for a service organization that is structurally equipped to work across silos if supported by appropriate collaborative governance mechanisms [15,166]. It has long been an adage of integrated care, as stated by Walter Leutz, that “you can integrate all of the services for some of the people, some of the services for all of the people, but you can’t integrate all of the services for all of the people” [167 p.83]. Gaps in services are likely to be a feature of integrated care for at least some subset of the population. This study found that paramedic organizations partnered with a range of otherwise-siloed service providers in their geographic area, including hospitals, primary care, social services, community-based allied health such as nursing and physiotherapy, mental health facilities and homeless shelters. The role of the paramedic organization can be conceptualized as maintaining a mobile logistics infrastructure, a 24/7 staff of a flexible, generalist workforce and service agreements with agencies in their catchment area. These agreements extend and expand the reach of partner agencies in terms of out-of-hours coverage, unscheduled response and mobility in the community. There may be value in having such a ‘gap-filling’ organization of providers that is able to work across the inevitable silos in the system. However, this organization may benefit from a loose collaborative governance structure with health and social organizations in their geographic area, perhaps in the form of a formal body that regularly meets to identify new gaps in care and resolve challenges. It would be necessary to have a clearly articulated mandate or strategic framework and a shared understanding of the function of this ‘gap-filling’ organization in the system to help direct partnerships so that they are more intentional [15].

### Need for permissive regulation

The role of legislation and regulation in integrated service delivery has been discussed in terms of the degree to which the authority of governments is used to impose change versus a softer, ‘hands off’ approach that fosters integration [11,12]. This study found that programs faced challenges adapting their service offerings to address local needs due to legal and regulatory constraints. For instance, some programs involving paramedics faced challenges with medicolegal oversight, a narrowly defined scope of practice and a requirement to transport all clients to hospitals. Broadly this speaks to the need for laws and regulations to be less prescriptive in defining the actions of care providers as it may limit local systems from designing care pathways and service offerings that are most appropriate for their population and contexts. Legal frameworks could instead seek to be permissive, allowing local changes in practice, while still ensuring appropriate oversight and safety mechanisms. In a sense, they could provide a sandbox in which local systems can play rather than directing the actions of providers. Others have suggested this can create unwanted care variation and that standardization is a way to ensure care equity [15], but this is likely a false dichotomy. As seen with New Zealand’s ongoing experience with their System Level Framework for driving local health system improvement [168], governance and regulation could focus on establishing broad, negotiable performance measures and actively managing the process of service design, rather than prescribing activity and scope. This may strike a better balance between ensuring safety and service equity at the system level, while enabling integration at the local level which can be constrained by an overly standardized legal framework.

### Assessing value at a systems level

Consistent with challenges highlighted by previous studies on payment models for integrated care [169,170], the programs in this study expressed the need to assess cost-effectiveness and value at a systems level. In many examples, services were paid for through service-specific budgets or a grant specific to an initiative [51,94]. There were a few examples where a capitated budget for a target population was used, or resources were shared between organizations and care teams [115,129,155]. Some programs, particularly in the UK, compared the cost-effectiveness of different clinical pathways by combining per-unit costs of multiple services in a chain of care [79,100]. However, much of the literature suggested that funding models were misaligned and failed to capture the value of new programs and services. For instance, the value of cost savings in other parts of the system such as avoided emergency costs from effective prevention [95], or the value of better client experience from more appropriate care pathways. Typically, payment models for integrated care depend on factors such as complexity of client needs and the duration of services (e.g., ongoing versus episodic) [171]. In this study, the same providers – paramedics – were providing episodic and ongoing services to low- and high-needs populations through geographically-based cross-cutting organizations. Paying for this type of service may involve mixing payment mechanisms for different service types, including base funding, capitation and fees-for-service. The way to fund these services sustainably and assess their value to the broader system requires further study.

### Limitations

This study has several limitations that relate to: (a) approach to the literature search; (b) types of literature available; and (c) international applicability. This study was exploratory in nature as the role of paramedics in integrated care is a new area of research. As such, the literature search and keywords were designed based on the study team’s knowledge and consultation with experts; it is possible that some models of care were missed due to the assumptions embedded in study design. For example, no literature from Sweden was included in the analysis as the documents from Sweden only focussed on the role of nurses who work on ambulances, and thus did not meet the inclusion criteria of functions performed by paramedics. This assumption of paramedicine as a unique profession was embedded in the research design, search keywords and literature screening. Secondly, over 25% of literature was not peer-reviewed and came from trade journals and magazines, and a majority of peer-reviewed literature was comprised of case reports and observational studies. This is consistent with other recent reviews [43] and reflects the current state of academic discourse in paramedicine. Finally, all the programs included in the analysis were from high-income westernized nations in Europe, North America and Australasia; this may limit the applicability of findings to low- and middle-income countries.

## Conclusion

This study adds to the literature that integrated care can be supported by a flexible, generalist health workforce, local organizations that work across silos and legislation that balances standardization with flexibility. Through studying integrated care in the paramedic context, this study found that paramedics are often bridging gaps between acute and chronic care for a broad range of populations; playing a case finding role within health systems; and serving as additional human resource for public health initiatives. This suggests that connectivity and collaboration in local care systems can be enhanced by cross-cutting organizations with a generalist workforce that extends the reach of existing services in a geographic area through a single point-of-care. However, further work is needed to determine the appropriate skill mix of professionals to play these roles, on addressing differences in professional norms, and regulation and payment mechanisms to support such services. The highly localized programs analyzed in this study are often band-aids for fractured care systems in the absence of an enabling environment for integrated care. The findings of this study can help health system leaders address these gaps in a more systematic and intentional way.

## Additional Files

The additional files for this article can be found as follows:

10.5334/ijic.6418.s1 Supplemental File 1. Sample search query.

10.5334/ijic.6418.s2 Supplemental File 2. List of 137 citations included in final analysis and descriptions of programs.
