# Supplementary material for: Designing and Governing Responsive Local Care Systems – Insights from a Scoping Review of Paramedics in Integrated Models of Care
Source: Int J Integr Care. 2022 Apr 13;22(2):5. doi: 10.5334/ijic.6418 (PMC9009364; doi:10.5334/ijic.6418)
Supplement: Supplemental File 1. — Sample search query. [file ijic-22-2-6418-s1.pdf]

Supplemental File 1: Example database search query (Ovid MEDLINE)

	Searches
1	exp Emergency Medical Technicians/
2	emergency medical services/ or exp emergency medical dispatch/
3	(paramedic* or (emergency adj3 technician*) or (ambulance adj2 clinician*) or (ambulance adj2 personnel) or prehospital or EMS).tw,kf.
4	1 or 2 or 3
5	(paramedic* adj3 (communit* or advanced or practitioner* or extended or expanded)).tw,kf.
6	intersectoral collaboration/ or models, organizational/ or comprehensive health care/ or case management/ or exp "continuity of patient care"/ or exp patient-centered care/ or exp after-hours care/ or exp "delivery of health care, integrated"/ or managed care programs/ or patient care team/
7	(care adj4 (patient cent* or integrat* or coordinat* or community* or team)).tw,kf.
8	(model* adj3 (patient cent* or integrat* or coordinat* or community* or team)).tw,kf.
9	6 or 7 or 8
10	4 and 9
**11**	**5 or 10**
