# Supplementary material for: Designing and Governing Responsive Local Care Systems – Insights from a Scoping Review of Paramedics in Integrated Models of Care
Source: Int J Integr Care. 2022 Apr 13;22(2):5. doi: 10.5334/ijic.6418 (PMC9009364; doi:10.5334/ijic.6418)
Supplement: Supplemental File 2. — List of 137 citations included in final analysis and descriptions of programs. [file ijic-22-2-6418-s2.pdf]

Supplemental File 2: List of 137 citations included in analysis and description of models of care

Citation	Journal or Publication	Study Type	Peer Review	Country	Model of Care Name or Description	Target Population	Geographic and Operational Context	Services Provided
Abrashkin et al., 2016	Journal of the American Geriatrics Society	Observational Study	Yes	USA	Advanced Illness Management (AIM) program with community paramedicine	Adults with multiple chronic conditions such as CHF, COPD, Diabetes	Urban-suburban setting of Queens and Long Island, New York	Ongoing primary care, phone triage by nurse in case of exacerbation, home-based community paramedic visits if needed with treatment at home
Abrashkin et al., 2019	BMJ Supportive & Palliative Care	Observational Study	Yes	USA	Physician extender community paramedic model within an advanced illness management (AIM) program	Adults with multiple chronic conditions such as CHF, COPD, Diabetes	Queens, Nassau, and Suffolk counties, New York, USA	Home-based primary care, on-demand assessment and treatment for acute and urgent needs through appointments, paramedic visit and treatment at home, or referral and transport to ED

Citation	Journal or Publication	Study Type	Peer Review	Country	Model of Care Name or Description	Target Population	Geographic and Operational Context	Services Provided
Agarwal et al., 2019	Prehospital Emergency Care	Experimental Study	Yes	Canada	Community Paramedicine at Clinic - CP@Clinic Program	Older adults living in the community	5 participating cities/municipalities in Ontario, Canada: Hamilton, York, Sudbury, Guelph, Simcoe	Weekly drop-in clinics in an apartment building with physical assessment, lifestyle- and quality of life assessment, recommendations for preventative care; reports to family physician for follow-up, referrals to support services to improve health behaviours
Allen, 2019	Emergency Nurse	Article or Vignette	No	UK	Mental Health Triage Hub	People experiencing mental health emergency	Derbyshire, UK	Support during acute mental health crisis, physical assessment, follow-up mental health care
Anastasio et al., 2015	EMS World	Article or Vignette	No	USA	EMS-based Mobile Integrated Healthcare (MIH)		Fort Worth, Texas and surrounding areas (USA)	Patient navigation and care coordination with primary care and home care during a medical emergency, with follow-up care in their home

Citation	Journal or Publication	Study Type	Peer Review	Country	Model of Care Name or Description	Target Population	Geographic and Operational Context	Services Provided
Antevy, 2017	EMS World	Article or Vignette	No	USA	Collaborative care model; full-risk managed services organization with mobile integrated health (MIH); ChanMed Model	Older adults living in the community	"Southwest Florida," "tri-county region (Palm Beach, Broward and Miami-Dade) and "six southeastern states" - USA	Primary care assessment, medication management, home care; in-home assessment, treatment, and follow-up in case of emergencies
Ashton et al., 2017	Healthcare Quarterly	Experimental Study	Yes	Canada	Community paramedicine home visit program (Ageing at Home)	Multiple	Two locations in Ontario, Canada: Renfrew County (rural) and Belleville in Hastings County (urban)	
BC Emergency Health Services, 2017		Government Document	No	Canada	Community paramedicine in British Columbia	People living in a defined rural area with limited access to services	76 rural and remote communities across British Columbia, Canada	Community outreach, health promotion, wellness (drop-in) clinics for specific health issues, wellness checks for older people who live alone
BC Emergency Health Services, 2018	Canadian Paramedicine	Article or Vignette	No	Canada	Community paramedicine	Multiple	89-99 rural and remote communities throughout the province of British Columbia	

Citation	Journal or Publication	Study Type	Peer Review	Country	Model of Care Name or Description	Target Population	Geographic and Operational Context	Services Provided
BC Emergency Health Services, 2019a	Canadian Paramedicine	Article or Vignette	No	Canada	Community paramedicine	People living in a defined rural area with limited access to services	99 rural and remote communities across the province of British Columbia, Canada	Home visits with care plan for specific patients; emergency response for entire community; health promotion and community outreach for entire community
BC Emergency Health Services, 2019b		Mixed Methods Study	No	Canada	Community paramedicine in british columbia	People living in a defined rural area with limited access to services	99 rural and remote communities across BC, Canada	In-home physical assessments, vital signs and wellness checks particularly for chronic disease (COPD, diabetes) and falls risk; health education and health promotion activities, including naloxone training, CPR, AED, car sear safety; palliative care; emergency response capacity in rural areas

Citation	Journal or Publication	Study Type	Peer Review	Country	Model of Care Name or Description	Target Population	Geographic and Operational Context	Services Provided
BCAS, 2014	Canadian Paramedicine	Article or Vignette	No	Canada	BC Ambulance Service Infant Transport Team (ITT)	Pediatric, neonatal, and high-risk obstetrics patients with complex needs	British Columbia, Canada. Based in Vancouver; serves hospitals throughout British Columbia, the Yukon Territories, other parts of Canada and the United States when required	Stabilization, treatment and transport to specialty care
Belt et al., 2016	Stroke	Observational Study	Yes	USA	In-Transit Telestroke (ITTS)	People with symptoms of stroke	Sub-urban New Jersey, USA	Identification, triage, and rapid treatment for stroke
Bennett et al., 2018	The Journal of Rural Health	Observational Study	Yes	USA	Community paramedicine program	Adults with multiple chronic conditions such as CHF, COPD, Diabetes	Abbeville County, South Carolina - a rural county on the western border of the state; population tends to be "less educated, more likely to live in poverty"	Home visits with health screening (falls, medications), referrals and coordination with community care and primary care services, care planning, enrollment in health insurance/financial support programs

Citation	Journal or Publication	Study Type	Peer Review	Country	Model of Care Name or Description	Target Population	Geographic and Operational Context	Services Provided
Boykin et al., 2018	American Journal of Health-System Pharmacy	Article or Vignette	Yes	USA	Community paramedic heart failure transition of care program; collaborative with Heart Strong program	People at high risk for hospital readmission	Wilmington, North Carolina, USA	Care coordination, medication reassessment and prescription assistance, symptom management, in-home lab tests and treatments to manage heart failure after discharge from hospital
Bronsky et al., 2016	Journal of Emergency Medical Services	Article or Vignette	No	USA	Community Response Team for Mental Health	People experiencing mental health emergency	Colorado Springs, Colorado	De-escalation and psychological support when in mental health crisis, medical clearance, transport and transfer of care to a behavioural health facility
Brydges et al., 2016	BMC Health Services Research	Qualitative Study	Yes	Canada	Community Health Assessment Program through Emergency Medical Services (CHAP-EMS) community paramedicine program	Multiple	Residents living in a subsidized housing building in a "large municipality in the Greater Toronto Area" in Ontario, Canada	Drop-in weekly health check-up, identification of health and social needs and individualized follow-up with relevant other services, identification of emergent issues and referrals to follow-up care, health advice

Citation	Journal or Publication	Study Type	Peer Review	Country	Model of Care Name or Description	Target Population	Geographic and Operational Context	Services Provided
Burns et al., 2013	European Journal of Emergency Medicine	Case Report or Case Study	Yes	Australia	Standard operating procedure for intra-aortic balloon pump (IABP) medical retrieval across road and rotary wing vehicles	Cardiac and intensive care patients requiring complex specialist interventions	Most of state of New South Wales in Australia, large geography of 809,444 km2, population of 7 million people	Ongoing advanced intensive and critical care during transit from one hospital to another
Bussieres et al., 2017	Journal of Telemedicine and Telecare	Case Report or Case Study	Yes	Canada	The Unite de Coordination Clinique des Services Prehospitaliers d'Urgence (UCCSPU: Emergency Prehospital Services Clinical Coordination Unit)	People living in a defined rural area with limited access to services	Chaudiere-Appalaches region of Quebec in Canada, sparsely populated rural region covering 15,073 km2.	Prehospital identification of heart attack (STEMI) and transfer to cardiac surgery (PCI); pain relief during emergencies; and other emergencies in a rural setting via emergency (paramedic) call or rural community health centre / emergency
Caid, 2016	Firehouse	Article or Vignette	No	USA	Community integrated paramedicine, mobile integrated healthcare	High users of the health or 911 system	Rio Rico, Arizona, USA. 1 hour south of the city of Tucson, 42-square-mile service area; also serve a "primary mutual aid" area of 119 square miles in neighbouring jurisdictions	Home visit, safety assessment, medication reconciliation, referral to community health resources

Citation	Journal or Publication	Study Type	Peer Review	Country	Model of Care Name or Description	Target Population	Geographic and Operational Context	Services Provided
Calderone et al., 2017		Article or Vignette	No	Canada	Community paramedicine	People living in a defined rural area with limited access to services	73 communities (pilot) in British Columbia, Canada	
Carter et al., 2019	Canadian Journal of Emergency Medicine	Mixed Methods Study	Yes	Canada	Paramedics Providing Palliative Care at Home	People in palliative care and their families	Nova Scotia and Prince Edward Island - two provinces in Canada	Medical and psychosocial support at end-of-life, including comfort measures, to facilitate dying at home
Chellappa et al., 2018	Internal Medicine Journal	Article or Vignette	Yes	USA	Community paramedicine program to support the visiting doctors program and hospitalization-at-home program	Adults with multiple chronic conditions such as CHF, COPD, Diabetes	New York City, USA	On-call telephone assessment by primary care physician in case of urgent unexpected medical problem, in-person assessment and treatment by paramedic if needed, recommendation of follow-up care (including emergency department if appropriate)
Chesters and Webb, 2015	European Journal of Emergency Medicine	Observational Study	Yes	UK	Doctor-paramedic prehospital care team on helicopter ambulance	People involved in major accident or trauma	Essex and Herts region in the UK	

Citation	Journal or Publication	Study Type	Peer Review	Country	Model of Care Name or Description	Target Population	Geographic and Operational Context	Services Provided
Chesters et al., 2013	Emergency Medicine Journal	Observational Study	Yes	UK	Helicopter-based doctor-paramedic prehospital care teams		Rural areas in the UK around Norfolk, Suffolk, Bedfordshire, Cambridgeshire	
Chesters et al., 2015	The Journal of Emergency Medicine	Observational Study	Yes	UK	Doctor-paramedic helicopter emergency medical service (HEMS)	People involved in major accident or trauma	Six counties in the East of England, mix of rural, urban, suburban areas	Advanced life support and post-resuscitation interventions after cardiac arrest or major trauma, transfer to PCI-capable hospital for coronary artery disease investigation and treatment
Comans et al., 2013	Injury Prevention	Case Report or Case Study	Yes	Australia	Referral by prehospital emergency services to a community-based falls-prevention service	Older adults that have had a fall	South-west area of Brisbane metropolitan area, Australia	Emergency assistance after a fall; referral and follow-up services: falls risk assessment at home, exercise, education, home hazard identification and modification

Citation	Journal or Publication	Study Type	Peer Review	Country	Model of Care Name or Description	Target Population	Geographic and Operational Context	Services Provided
Cooper et al., 2004	Emergency Medicine Journal	Mixed Methods Study	Yes	UK	Emerging role of Emergency Care Pracitioner (ECP)	People experiencing low-acuity emergencies	West Cornwall (predominantly rural) and West Devon (predominantly urban, in Plymouth), in Westcountry, UK	Assessment and treatment of emergencies, treatment on scene for some cases (e.g., wound closure), referral and coordination of follow-up care (via GP, community hospital, clinic, or emergency)
Cooper et al., 2007a	Emergency Medicine Journal	Case Report or Case Study	Yes	UK	Emergency care practitioner (ECP) role in the UK, "unscheduled out-of-hospital emergency care"	People experiencing low-acuity emergencies	Westcounty Region in the UK	Various services/roles played by ECPs in various settings; detailed list of services are unclear in this paper
Cooper et al., 2007b	see Cooper et al., 2007a	see Cooper et al., 2007a	Yes	UK	see Cooper et al., 2007a	see Cooper et al., 2007a	see Cooper et al., 2007a	see Cooper et al., 2007a
Cooper et al., 2008	International Emergency Nursing	Case Report or Case Study	Yes	UK	Emergency care practitioner (ECP) role in the UK NHS		UK Westcountry	Assessment, treatment, referral, and discharge for urgent and acute problems
Coyler et al., 2018	Air Medical Journal	Observational Study	Yes	USA	Pediatric critical care (interfacility) transport	Pediatric, neonatal, and high-risk obstetrics patients with complex needs	Regional referral centre for multiple states in the mid-western United States	Ongoing complex critical care and transport between facilities

Citation	Journal or Publication	Study Type	Peer Review	Country	Model of Care Name or Description	Target Population	Geographic and Operational Context	Services Provided
Crawford, 2016	Fire Rescue Magazine	Article or Vignette	No	USA	Community paramedicine; Community Care Specialist Unit	People experiencing low-acuity emergencies	Mesa, Arizona, USA	Emergency treatment and follow-up for "low medical acuity"
Creed et al., 2018	Prehospital Emergency Care	Observational Study	Yes	USA	Advanced Practice Paramedic (APP) Program - alternate destination program	People experiencing mental health emergency	Wake County, North Carolina, USA	On-call (emergency) assistance in mental health crisis, direct triage to crisis intervention, streamlined process to inpatient, outpatient, and addictions/detox services
Crockett et al., 2017	Journal of Pharmacy Practice	Case Report or Case Study	Yes	USA	Community Paramedicine Team	People at high risk for hospital readmission	Marion County in Indianapolis, Indiana	In-home assessment of physical and psychosocial needs, medication review, health education on managing heart failure, connections to social and financial supports via social worker
Dainty et al., 2018	Health Services Research	Qualitative Study	Yes	Canada	Expanding Paramedicine in the Community (EPIC) - community paramedicine program	Adults with multiple chronic conditions such as CHF, COPD, Diabetes	Two regions in Southwestern Ontario, Canada: York Region and Grey County	Scheduled home visits at 3-month intervals; follow-up visits as-needed; emergency home visits (24/7) in case of exacerbation

Citation	Journal or Publication	Study Type	Peer Review	Country	Model of Care Name or Description	Target Population	Geographic and Operational Context	Services Provided
Dieker et al., 2010	JACC: Cardiovascular Interventions	Observational Study	Yes	Netherlands	Prehospital triage for primary angioplasty in patients with STEMI	People with symptoms of heart attack	East of the Netherlands, parts of provinces of Gelderland and Brabant, radius of 48 miles	Emergency assessment, treatment, and transport to appropriate facility in case of heart attack, including timely evidence-based surgery (angioplasty)
Dissman and Clerc, 2007	Emergency Medicine Journal	Observational Study	Yes	UK	Helicopter emergency medical service	People involved in major accident or trauma	Cumbria (based in Penrith), and northeast region (based at Teesside), UK; mixed rural/urban cachement area	"Advanced medical interventions" such as rapid sequence intubation, advanced pain management, advanced trauma life support
Dixon et al., 2009	Emergency Medicine Journal	Experimental Study	Yes	UK	Paramedic Practitioner Older People's Support scheme	People experiencing low-acuity emergencies	South Yorkshire, UK - calls originating from a Sheffield postal code	Assessment and treatment for minor injuries and conditions at home with referrals to community care and follow-up services, as an alternative to standard paramedic + ED transport

Citation	Journal or Publication	Study Type	Peer Review	Country	Model of Care Name or Description	Target Population	Geographic and Operational Context	Services Provided
Doan et al., 2020	Prehospital Emergency Care	Observational Study	Yes	Australia	Prehospital field identification and treatment of ST-segment elevation myocardial infarction (STEMI)	People with symptoms of heart attack	Queensland, Australia; northeast part of Australia, 1.7 million km2, relatively high proportion of population living outside major urban centres (38% of population, compared to average of 23-27% in other places)	Emergency response, treatment, triage, and transport to appropriate specialty cardiac care in event of a heart attack
Dobson, 2014	Canadian Paramedicine	Article or Vignette	No	Canada	Two community paramedic programs: Collaborative Emergency Centres (CEC) and VISIT program	Multiple	Nova Scotia, Canada; 7 rural and remote hospitals (CECs), two rural communities (VISIT)	Emergency department in the overnight hours with in-depth assessment, management of emergent issues and care plan; in-home visit and assessment and follow-up from other health providers
Dorsch et al., 2008	American Heart Journal	Observational Study	Yes	UK	Direct ambulance admission to PCI after prehospital diagnosis of STEMI	People with symptoms of heart attack	A region in the UK with an urban population, with a number of peripheral hospitals and EDs and 1 cardiac centre with PCI capability	Rapid diagnosis, transport, and emergency treatment of sudden heart attack, and follow-up care from cardiac team

Citation	Journal or Publication	Study Type	Peer Review	Country	Model of Care Name or Description	Target Population	Geographic and Operational Context	Services Provided
Ebinger et al., 2015	JAMA Neurology	Experimental Study	Yes	Germany	Stroke Emergency Mobile Unit (STEMO) part of the Prehospital Acute Neurological Treatment and Optimization of Medical Care in Stroke (PHANTOM-S) study	People with symptoms of stroke	Urban area of Berlin, Germany	Emergency response, diagnostics (CT, lab work), and treatment for acute stroke in the field; transport and hand-off to follow-up care
Enrich, 2013a	EMS World	Article or Vignette	No	USA	Community paramedic initiative	Multiple	Pittsburgh and it's neighbouring suburbs, in the state of Pennsylvania, USA	
Enrich, 2013b	EMS World	Article or Vignette	No	USA	Mobile integrated health for CHF	People at high risk for hospital readmission	Chicago, USA	Post-discharge home care for CHF, symptom and wireless vital signs monitoring, 24/7 support from care team, health education, medication management

Citation	Journal or Publication	Study Type	Peer Review	Country	Model of Care Name or Description	Target Population	Geographic and Operational Context	Services Provided
Epton et al., 2018	BMJ Open Respiratory Research	Article or Vignette	Yes	New Zealand	New service model for exacerbations of chronic obstructive pulmonary disease (COPD)	Adults with multiple chronic conditions such as CHF, COPD, Diabetes	Christchurch, New Zealand	Emergency treatment and discharge or appropriate follow-up care from acute, community-based, GP or emergency departments for an exacerbation of COPD. Care processes customized to the patient's goals of care.
Faddy et al., 2017	Australasian Psychiatry	Case Report or Case Study	Yes	Australia	Mental Health Acute Assessment Team	People experiencing mental health emergency	Western Sydney, Australia; 780 km2	Physical and mental health assessment during an emergency, triage to ED, mental health facility, or discharge with appropriate follow-up care (inpatient or outpatient)
Fischer et al., 2020	Journal of Substance Use	Case Report or Case Study	Yes	USA	Sobering centre, alternative destination, harm reduction	People who are currently intoxicated	South King County, Washington, USA; "a high-need and under-resourced area south of Seattle" including the Cities of Kent and Renton	Medically-monitored space to become sober in case of alcohol intoxication; referral to and connection with follow up social and addictions services

Citation	Journal or Publication	Study Type	Peer Review	Country	Model of Care Name or Description	Target Population	Geographic and Operational Context	Services Provided
Georgiev et al., 2019	The Journal for Nurse Practitioners	Case Report or Case Study	No	USA	Community paramedicine transitional health support program	People at high risk for hospital readmission	Urban neighbourhood in an unidentified city in the state of Maryland, USA	Case management, in-home community-based services, comprehensive assessment during a home visit, home safety
Geskey et al., 2020	Population Health Management	Observational Study	Yes	USA	Post-discharge intervention by EMS	People at high risk for hospital readmission	Specific zip code in Hilliard, Ohio, USA - Norwich Township - outskirts of Columbus	Home visit after being discharged from hospital to go over discharge instructions, assessment of patient's physical health, identify gaps in care, connections to and facilitation of community services
Heinelt et al., 2015	Prehospital Emergency Care	Case Report or Case Study	Yes	Canada	Community paramedicine - Expanding Paramedicine in the Community (EPIC) trial	Adults with multiple chronic conditions such as CHF, COPD, Diabetes		Regular home visits with physical, social, environmental assessments; follow-up by primary care teams

Citation	Journal or Publication	Study Type	Peer Review	Country	Model of Care Name or Description	Target Population	Geographic and Operational Context	Services Provided
Hertig et al., 2017	Currents in Pharmacy Teaching and Learning	Observational Study	Yes	USA	Community Paramedic Transition of Care Program	People at high risk for hospital readmission		Home visit after discharge from hospital to identify emergent symptoms, reinforce discharge instructions, provide health education, physical assessment and coordination with primary care providers, medication review
Hoyle et al., 2012	Emergency Medicine Australasia	Observational Study	Yes	New Zealand	Extended Care Paramedic (ECP) model in New Zealand	People experiencing low-acuity emergencies	Kapiti district, 50-60 km north of Wellington, New Zealand, where closest ED is	In-home assessment, treatment, discharge, referral to follow-up services, referral to ED
Iezzoni et al., 2018	The American Journal of Managed Care	Observational Study	Yes	USA	Acute Community Care Program	All people rostered with a primary care practice or organization	Eastern Massachusetts, USA	Phone assessment and triage by a nurse, urgent care provided in the home (blood work, assessment, treatment) by paramedics, follow-up care or transfer to ED if necessary
Iezzoni et al., 2019	American Journal of Medical Quality	Case Report or Case Study	Yes	USA	Acute Community Care Program	All people rostered with a primary care practice or organization	Massachusetts, USA	

Citation	Journal or Publication	Study Type	Peer Review	Country	Model of Care Name or Description	Target Population	Geographic and Operational Context	Services Provided
Jensen et al., 2013	Canadian Journal of Emergency Medicine	Observational Study	Yes	Canada	Extended care paramedic program (ECP) in long-term care (LTC) facilities	People in long-term care	Halifax, Nova Scotia, Canada	Assessment and treatment for minor and urgent conditions on-site at LTC with scheduled follow-up care as required
Jensen et al., 2014	Prehospital Emergency Care	Qualitative Study	Yes	Canada	Extended Care Paramedic program for long-term care	People in long-term care	Nova Scotia, Canada	Treatment and assessment at home (in long-term care) for acute and urgent conditions, scheduled follow-up at ED or at home, end-of-life care and counciling on end-of-life decision making for families
Jensen et al., 2015	Prehospital Emergency Care	Observational Study	Yes	Canada	Extended Care Paramedic program for long-term care, "Care by Design"	People in long-term care	Halifax, Nova Scotia, Canada	Assessment and treatment of acute or urgent conditions in the long-term care home, referral and follow-up care as-need by physicians and EDs
Johnson, 2015	Modern Healthcare	Article or Vignette	No	USA	Community paramedicine	High users of the health or 911 system	Fort Worth, Texas, USA	Home visit with medical assessment, care plan, follow-up, assistance with additional services as-neeeded

Citation	Journal or Publication	Study Type	Peer Review	Country	Model of Care Name or Description	Target Population	Geographic and Operational Context	Services Provided
Joy et al., 2019	Emergency Medicine Journal	Observational Study	Yes	UK	Physician Response Unit	Patients seeking emergency treatment for specific conditions where treatment can be safely provided at home	the borough of Tower Hamlets, "but also responding within Newham, City and Hackney, Waltham Forest, and beyond" - in London, UK	Emergency response for acute and urgent care with full diagnostic testing, assessment, and treatments at home or in the community. Follow up by connection to follow-up care in either the community (primary or specialty care, follow-up testing, physical/occupational therapy, district nursing, social work) or onward transport to the ED for additional immediate care
Karrer et al., 2015	Journal of Emergency Medical Services	Article or Vignette	No	USA	Community paramedicine	High users of the health or 911 system	Montgomery County, Texas Hospital District, Texas, USA	Comprehensive assessment and needs identification, navigation and care planning, care coordination and case management

Citation	Journal or Publication	Study Type	Peer Review	Country	Model of Care Name or Description	Target Population	Geographic and Operational Context	Services Provided
Klich, 2014	Canadian Paramedicine	Article or Vignette	No	Canada	Toronto EMS Community Paramedicine Program: Community Referral by EMS (CREMS) and Community Agency Notification (CAN)	Older adults living in the community	City of Toronto, Ontario, Canada.	Connecting patients with support agencies; keeping patient's support networks connected as they transition through the health care system
Knowles et al., 2011	Emergency Medicine Journal	Observational Study	Yes	UK	Paramedic Practitioner in Older People's Support (PPOS) scheme	People experiencing low-acuity emergencies	South Yorkshire, UK	
Kummer et al., 2018	Applied Clinical Infomatics	Case Report or Case Study	Yes	USA	Mobile Stroke Unit (MSU)	People with symptoms of stroke	Two neighbourhoods in New York City that are associated with University Medical Centres: Upper East Side and Washington Heights	Assessment, CT scan, and rapid treatment for stroke followed by transport to hospital
Kusel and Savino, 2015	Journal of Emergency Medical Services	Article or Vignette	No	USA	Mobile Integrated Healthcare and Community Paramedicine (MIH-CP)	People at high risk for hospital readmission	Alameda County, California, USA	In-home assessment, case management, health service navigation

Citation	Journal or Publication	Study Type	Peer Review	Country	Model of Care Name or Description	Target Population	Geographic and Operational Context	Services Provided
Lamhaut et al., 2013	Resuscitation	Observational Study	Yes	France	Mobile Intensive Care Unit (MICU)	People experiencing sudden cardiac arrest	Paris, France	Full ACLS care for cardiac arrest and ICU-level care (blood products, diagnostics including ultrasounds, and ECLS) in the field
Langabeer II et al., 2016	Western Journal of Emergency Medicine	Observational Study	Yes	USA	Emergency Telehealth and Navigation (ETHAN) Program	People experiencing low-acuity emergencies	City of Houston in Texas, USA; urban, 600 square miles	On-call emergency medical assessment by calling 911, telemedicine assessment with physician over video call, triaged to ED via ambulance; transport to local clinic, ED, primary care physician including via taxi service; phone aftercare instructions
Lau et al., 2018	Prehospital Emergency Care	Qualitative Study	Yes	USA	Community paramedicine care transitions intervention	People at high risk for hospital readmission	Madison, Wisconsin and Rochester, New York	
Le May et al., 2006	The American Journal of Cardiology	Observational Study	Yes	Canada	Paramedic-diagnosed STEMI with early transport to primary PCI centre	People with symptoms of heart attack	City of Ottawa, Ontario, Canada; metropolitan area, approx 53 km radius	Emergency diagnosis, transport, supportive treatment and definitive treatment (pharmaceutical and surgery) in case of a sudden heart attack

Citation	Journal or Publication	Study Type	Peer Review	Country	Model of Care Name or Description	Target Population	Geographic and Operational Context	Services Provided
Le May et al., 2012	Journal of the American College of Cardiology	Observational Study	Yes	Canada	Direct transport/triage of patients to primary PCI centres, STEMI system of care	People with symptoms of heart attack	City of Ottawa, Canada	Cardiac assessment, electrocardiogram in the field, rapid triage and direct transport to a PCI-capable centre, angiography and angioplasty, followed by rehab/recovery post-surgery
Lerner et al., 2003	American Journal of Emergency Medicine	Case Report or Case Study	Yes	USA		People with diabetes experiencing an acute hypoglycemic episode		Treatment of hypoglycemia on scene, provision of discharge instructions for follow-up care
Liem et al., 2007	American Heart Journal	Observational Study	Yes	Netherlands	MISSION! Protocol for all-phase integrated acute myocardial infarction care program	People with symptoms of heart attack	Hollands-Madden Region, approx 50x25 miles	Emergency and acute diagnosis and treatment, surgery (as needed), post-op, rehabilitation, health education and follow-up primary care for one year after a heart attack
Machen et al., 2007	Accident and Emergency Nursing	Qualitative Study	Yes	UK	Pilot service new response to low-priority ambulance calls	People experiencing low-acuity emergencies	Defined geographical area, East of England, UK	Assessment and treatment at home for low-acuity emergencies, including wound dressing, suturing, catheter care

Citation	Journal or Publication	Study Type	Peer Review	Country	Model of Care Name or Description	Target Population	Geographic and Operational Context	Services Provided
Mackey and Qiu, 2018	Prehospital Emergency Care	Observational Study	Yes	USA	Mobile integrated health care paramedic to safely conduct behavioural clearance of mental health patients	People experiencing mental health emergency	Stanislaus County in central California, which has two large population centres	Field-triage and medical clearance to be allowed direct transport, assessment, or admission to psychiatric services; acute, inpatient and outpatient mental health services
Marshall et al., 2015	Canadian Family Physician	Case Report or Case Study	Yes	Canada	Care by Design - a new model of care for long-term care facilities	People in long-term care	Halifax, Nova Scotia, Canada	Regular on-site visits from family physician, 24/7 on-call physician coverage, on site acute care and coordinated transfer to ED if needed, comprehensive geriatric assessment
Martin and O'Meara, 2019	Rural and Remote Health	Qualitative Study	Yes	Canada & USA	Community paramedicine	People living in a defined rural area with limited access to services	Two different rural areas: a county in Ontario, Canada with 17 municipalities across 4,969 km2; a paramedic service in Colorado, USA serving 12 rural communities across 2,723 km2	Home visits and wellness checks, response for urgent non-emergency needs, community-based prevention and educational services

Citation	Journal or Publication	Study Type	Peer Review	Country	Model of Care Name or Description	Target Population	Geographic and Operational Context	Services Provided
Martin et al., 2016	The Australian Journal of Rural Health	Qualitative Study	Yes	Canada	Community paramedicine in rural area	People living in a defined rural area with limited access to services	A county in rural Ontario, Canada encompassing 17 municipalities spread over 8,000 km2	
Martin-Misener et al., 2009	Primary Health Care Research & Development	Mixed Methods Study	Yes	Canada	Nurse practitioner-paramedic-family physician model of care in a rural area	People living in a defined rural area with limited access to services	Long and Brier Islands, a geographically remote area in Nova Scotia, Canada; accessible by a boat; the islands do not have a resident physician	Primary care, simple wound care, immunizations, home assessments, lifestyle counseling for prevention and health promotion: smoking cessation and weight loss; fitness, wellness services; home visitation services for isolated elderly; illness and injury prevention; health screening for breast cancer, depression, bone density, falls prevention

Citation	Journal or Publication	Study Type	Peer Review	Country	Model of Care Name or Description	Target Population	Geographic and Operational Context	Services Provided
Mason et al., 2003	Emergency Medicine Journal	Article or Vignette	Yes	UK	Community paramedic practitioner intermediate care support scheme	Older adults living in the community		Emergency assessment and treatment of an urgent problem at home or in the community, with follow-up testing and treatment as-needed arranged at the appropriate facility (ED, clinic, community services)
Mason et al., 2007	British Medical Journal	Experimental Study	Yes	UK	Paramedic Practitioner in Older People's Support Scheme (PPOPS)	Older adults living in the community	South Yorkshire, UK; calls originating from a Sheffield postal code (one large urban area)	In-home assessment, treatment and referral to follow-up care if needed for low-acuity emergencies. Services include wound care, suturing, splintage, thorough examination of joints, respiratory, cardiovascular, neurological, ENT; protocol-led dispensing including antibiotics. Urine and blood tests, eligibility for radiology, referral to additional services

Citation	Journal or Publication	Study Type	Peer Review	Country	Model of Care Name or Description	Target Population	Geographic and Operational Context	Services Provided
Mason et al., 2012	Emergency Medicine Journal	Experimental Study	Yes	UK	Emergency Care Practitioner (ECP) role within the NHS	People experiencing low-acuity emergencies	Multiple sites in England and Scotland	Primary care consultation, diagnostics, treatment, and referral to follow-up services
McDonald and Sneath, 2018	Canadian Paramedicine	Article or Vignette	No	Canada	Community paramedicine in Winnipeg: Main Street Project, Emergency Paramedic in the Community (EPIC), and At-Risk Referral (ARR)	Multiple	Winnipeg, Manitoba, Canada	On-site urgent and primary care and care coordination for clients of the shelter; home visits and case management for frequent users of 911 system; home assessment and referral to home care for clients deemed "at risk" after an emergency 911 call
McQueen et al., 2013	Emergency Medicine Journal	Observational Study	Yes	UK	Medical Emergency Response Incident Team (MERIT) as part of the West Midlands Major Trauma Network	People involved in major accident or trauma	Mixed urban and rural geographic area with one metropolitan area, several small towns, and some isolated rural communities	Intensive/critical-care level interventions in case of acute medical emergency or major trauma

Citation	Journal or Publication	Study Type	Peer Review	Country	Model of Care Name or Description	Target Population	Geographic and Operational Context	Services Provided
Mettner, 2013	Minnesota Medicine	Article or Vignette	No	USA	Community paramedics in Minnesota	Multiple	Multiple communities in the state of Minnesota, USA	Case management, assessment, home visits with regular coaching on things like medication compliance, blood work and diagnostic testing, referrals to support services like food and nutrition
Mikolaizak et al., 2016	Age and Ageing	Experimental Study	Yes	Australia	Intervention to PREvent Falls After Emergency Response (iPREFER) RCT	Older adults that have had a fall	Cluster of seven ambulance stations in the suburbs of Eastern Sydney, Australia	Exercise therapy, home medication review, home hazard assessment, vision assessment, geriatric assessment, transportation to outpatient or clinic appointment if needed

Citation	Journal or Publication	Study Type	Peer Review	Country	Model of Care Name or Description	Target Population	Geographic and Operational Context	Services Provided
Misner, 2005		Article or Vignette	No	Canada	Community paramedicine in rural island community	People living in a defined rural area with limited access to services	Two isolated island communities of Long and Brier, accessible via ferry, 30-50 minutes from Digby, Nova Scotia, Canada	24/7 emergency paramedic coverage, flu shots and drop-in clinics; diabetes checks, CHF assessment, antibiotics, urinanalysis, medication compliance assessment; specific services for individual patients requiring wound care (including suture/staple removal), falls prevention, health education, phlebotomy
Mitchell, 2018	Nursing Times	Article or Vignette	No	UK	Mental health team ambulance scheme	People experiencing mental health emergency	South-east London, UK	Mental health assessment, brief psychological interventions, physical assessment, follow-up care as-needed
Municipality of Chatham-Kent, 2019		Government Document	No	Canada	Paramedic-led wellness clinic	Older adults living in the community	Pilot site - one commuty housing building in Chatham, Ontario	Free weekly clinic session in the building with health check, referrals to preventative health services

Citation	Journal or Publication	Study Type	Peer Review	Country	Model of Care Name or Description	Target Population	Geographic and Operational Context	Services Provided
Nejtek et al., 2017	American Journal of Emergency Medicine	Case Report or Case Study	Yes	USA	Mobile Integrated Health program (MIH)	High users of the health or 911 system	Part of Tarrant County, Texas, USA ("North Texas")	In-home assessments, preventative care advice, psychosocial support, referrals to community-based services
O'Hara et al., 2019	BMJ Quality & Safety	Qualitative Study	Yes	UK	Telephone advice by Emergency Medical Services	People experiencing low-acuity emergencies	Multiple regional ambulance services across the UK; large geographical areas with mix of urban and rural settings	Telephone assessment and triage, self-care advice, referral to follow-up services or emergency ambulance
O'Meara et al., 2012	Rural and Remote Health	Case Report or Case Study	Yes	Australia	Rural Expanded Scope of Practice paramedic roles (RESP)	People living in a defined rural area with limited access to services	Four small, isolated, rural areas in Australia: Bordertown in South Australia; Scamander in northeastern Tasmania; Coleambally and Burham in New South Wales; Omeo and Mallacoota in Victoria	

Citation	Journal or Publication	Study Type	Peer Review	Country	Model of Care Name or Description	Target Population	Geographic and Operational Context	Services Provided
O'Meara et al., 2015	Australasian Journal of Paramedicine	Qualitative Study	Yes	Canada	Community paramedicine program with four components: Ageing at Home Program, Wellness Clinics, Ad-Hoc Home Visiting, and Community Paramedic Response Unit		Renfrew County - rural county in Ontario, Canada	
O'Meara et al., 2016	BMC Health Services Research	Case Report or Case Study	Yes	Canada	Community paramedicine		Renfrew County, Ontario, Canada	
Pearson and Shaler, 2017	Journal of Health and Human Services Administration	Case Report or Case Study	Yes	USA	Community paramedicine	Multiple	Rural and urban areas of Androscoggin County, Maine, USA; based and Lewiston. Two urban centres, with 45% of population in rural areas.	Home visits, wellness checks and screening (home safety, fall prevention), wound care, flu vaccination, medication reconcilliation, blood draws

Citation	Journal or Publication	Study Type	Peer Review	Country	Model of Care Name or Description	Target Population	Geographic and Operational Context	Services Provided
Pennel et al., 2016	Journal of Health Care for the Poor and Underserved	Case Report or Case Study	Yes	USA	EMS-based care coordination	Multiple	Three different rural communities in the state of Texas, USA. Each has service areas of between 500 and 1000 square miles.	Care coordination, including navigating services such as: applying for disability or financial assistance, filling prescriptions, referrals to primary and specialty care, home safety inspection and installation of assistive devices
Phelan et al., 2016	Frontiers in Public Health	Mixed Methods Study	Yes	USA	Fall prevention intervention at-scene by EMTs	Older adults that have had a fall	King County, Washington, USA; 50 square-miles	Education and advice, health promotion, referral to follow-up care RE fall prevention
Proctor, 2019	Journal of Paramedic Practice	Qualitative Study	Yes	UK	Paramedic practitioner role in GP practices		Various GP practices in the UK	Home visits for primary care problems

Citation	Journal or Publication	Study Type	Peer Review	Country	Model of Care Name or Description	Target Population	Geographic and Operational Context	Services Provided
Pruett et al., 2018	Journal of Emergency Medical Services	Article or Vignette	No	USA	Mobile integrated health / community paramedicine	People living in a defined rural area with limited access to services	Laguna Pueblo, federally-recognized Native American tribe, 45 miles west of Albuquerque, New Mexico, USA. rural "frontier", "geographically and culturally isolated", 825 square miles, population density of 4.9 people per square mile	Wound care for chronic wounds; medication management
Roberts et al., 2009	Emergency Medicine Journal	Observational Study	Yes	UK	Helicopter emergency medical service, doctor-paramedic unit	People involved in major accident or trauma	The Midlands, United Kingdom. Specifically: Warwickshire, Northamptonshire, West Midlands, Herefordshire, Gloucestershire, Worcestershire, Shropshire, Staffordshire, and the East Midlands	Advanced life support, airway, and surgical interventions before and during transport to hospital after an acute emergency, particularly major trauma

Citation	Journal or Publication	Study Type	Peer Review	Country	Model of Care Name or Description	Target Population	Geographic and Operational Context	Services Provided
Sampson et al., 2017	Diabetes Research and Clinical Practice	Observational Study	Yes	UK	Integrated care pathway for ambulance attended severe hypoglycemia in the East of England - single point-of-contact model (SPOC)	People with diabetes experiencing an acute hypoglycemic episode	East of England, a 7500 square mile area in the UK: Norfolk, Suffolk, Cambridgeshire, Bedfordshire, Hertfordshire, and Essex	Acute severe hypoglycemia management and follow-up: education program on hypoglycemia management, coordination with primary care and diabetes care services to enable reassessment
Savage et al., 2014	Heart, Lung and Circulation	Observational Study	Yes	Australia	Prehospital notification and initiation of treatment for STEMI	People with symptoms of heart attack	Queensland area in Australia, metropolitan	Emergency treatment and direct transport and transfer to specialty team in case of a heart attack (STEMI)
Schaefer et al., 2002	Prehospital Emergency Care	Observational Study	Yes	USA	Alternate destination of patient care	People experiencing low-acuity emergencies	Urban/suburban in the Greater Seattle Area	Emergency treatment and transport to hospital, triage and transport to primary/urgent care clinic

Citation	Journal or Publication	Study Type	Peer Review	Country	Model of Care Name or Description	Target Population	Geographic and Operational Context	Services Provided
Scharf et al., 2019	Prehospital and Disaster Medicine	Observational Study	Yes	USA	Mobile integrated community health	High users of the health or 911 system	Queen Anne's County, Maryland, USA - rural jurisdiction spanning 372 square miles, density 128.5 people per square mile; no hospital in jurisdiction but freestanding ED exists	Home visits from providers, health education and navigation to relevant services, information on how to advocate for their health, basic medical assessment at home and home safety assessment
Shah et al., 2010	Journal of the American Geriatrics Society	Case Report or Case Study	Yes	USA		Older adults living in the community	Livingston County, a rural county in New York state, USA. It has one small community hospital and 59 physicians; 12 different agencies providing EMS services, 11 of them volunteer and 1 with paid staff	Screening and referral for social services, particularly for depression, falls, and medication management; in-depth at-home assessment and referral / connections to follow-up services to address unmet needs

Citation	Journal or Publication	Study Type	Peer Review	Country	Model of Care Name or Description	Target Population	Geographic and Operational Context	Services Provided
Shah et al., 2018	Journal of the American Geriatrics Society	Observational Study	Yes	USA	Community Paramedic Care Transitions Intervention	People at high risk for hospital readmission	Three EDs across two medium-sized cities in Monroe County, New York (2 EDs) and Dane County, Wisconsin (1 ED)	Coaching (1 in person home visit and up to 3 phone calls) after being discharged from an ED on follow-up care instructions, medication management, health education on their conditions
Siddle et al., 2018	American Journal of Emergency Medicine	Observational Study	Yes	USA	Mobile integrated health - transition of care program	People at high risk for hospital readmission	One county in Indianapolis, Indiana, USA	Post-discharge home visit with structured assessment of physical, home environment, social, financial needs. follow-up phone calls and visits as-needed
Sinclair and Werman, 2009	Air Medical Journal	Observational Study	Yes	USA	Regional critical care transport service	Cardiac and intensive care patients requiring complex specialist interventions	Central and southeastern Ohio, USA	Full ICU-level advanced interventions (equipment, airways, medication infusion) during transport from one facility to another

Citation	Journal or Publication	Study Type	Peer Review	Country	Model of Care Name or Description	Target Population	Geographic and Operational Context	Services Provided
Smith et al., 2019a	Emergency Medicine Journal	Observational Study	Yes	UK	Enhanced care team, helicopter ambulance service	People involved in major accident or trauma	North East and North West England, 8,000 square miles	Critical care interventions on scene of emergency and during transport to hospital via ground or air, including: intubation with drugs, blood product administration (red blood cells, plasma), thoracotomy
Smith et al., 2019b	Forum	Article or Vignette	No	UK	Community paramedicine	People living in a defined rural area with limited access to services	Four pilot areas in Northern Ireland and South West Scotland	Full physical assessment, medications and treatments for various conditions (chronic exacerbations and acute, minor injuries), catheter maintenance, referrals and follow-up care from primary care teams,

Citation	Journal or Publication	Study Type	Peer Review	Country	Model of Care Name or Description	Target Population	Geographic and Operational Context	Services Provided
Snooks et al., 2017	Annals of Emergency Medicine	Experimental Study	Yes	UK	Support and Assessment for Fall Emergency Referral (SAFER) 2 trial	Older adults that have had a fall	Geographic operating area of 3 ambulance services in the UK, cluster-randomized trial by paramedic station	Immediate assistance and assessment after a fall; physical examination to potentially rule out emergent need; referral and follow-up with a falls service (and remaining home) if eligible and consenting
Stevens and Weinstein, 2013	Relias Media	Article or Vignette	No	USA	Treat the Streets: Pre-Hospital Pediatric Asthma Intervention Model to Improve Child Health Outcomes	Children with asthma	"narrowly focussed on a single county in Indiana" specifically targeting patients of the Riley Hospital for Children in Indianapolis	One comprehensive home assessment which can trigger additional community-based follow-up services after discharge from hospital or ED
Stirling et al., 2007	Rural and Remote Health	Qualitative Study	Yes	Australia	Rural expanded scope paramedics (ESP)	People living in a defined rural area with limited access to services	Multiple rural areas in Australia, but this study focussed on four cases in the Australian states of: Tasmania, New South Wales, South Australia, and Victoria	

Citation	Journal or Publication	Study Type	Peer Review	Country	Model of Care Name or Description	Target Population	Geographic and Operational Context	Services Provided
Swain et al., 2012	Emergency Medicine Australasia	Observational Study	Yes	New Zealand	Extended Care Paramedic (ECP) as part of an Urgent Community Care (UCC) pilot scheme	Older adults living in the community	Kapiti district, outside of Wellington, New Zealand	Paramedic assessment, diagnostics, referral to follow-up care, transport to GP or hospital if necessary
Tangherlini et al., 2016	Prehospital and Disaster Medicine	Observational Study	Yes	USA	Homeless Outreach and Medical Emergency (HOME) Team	High users of the health or 911 system	San Francisco, California	Connection and referral to community-based services: case management, social work, substance abuse and mental health treatment programming, primary care
Travers, 2018	Healthcare Management Forum	Article or Vignette	Yes	Canada	Emergency health service system in Nova Scotia	Multiple	Province of Nova Scotia, Canada	
van 't Hof, 2006	American Heart Journal	Experimental Study	Yes	Netherlands	Prehospital diagnosis, triage, and therapy for acute myocardial infarction (STEMI) - the On-TIME Study	People with symptoms of heart attack	Zwolle, The Netherlands	Emergency assessment, diagnosis, and evidence-based treatment for heart attack including transport and triage to cardiac catheterization

Citation	Journal or Publication	Study Type	Peer Review	Country	Model of Care Name or Description	Target Population	Geographic and Operational Context	Services Provided
Verma et al., 2018	Prehospital Emergency Care	Observational Study	Yes	Canada	Community Referrals by Emergency Medical Services (CREMS)		Toronto, Ontario - a major metropolitan city in Canada	During a 9-1-1 call (emergency event), referral to home-based nursing, physical and occupational therapy, personal support worker, social work, dietician, and care coordination services
Villarreal et al., 2017	Emergency Medicine Journal	Observational Study	Yes	UK	New model of service, GP partnership with ambulance service	People experiencing low-acuity emergencies	Worcestershire, mixed urban-rural county in the UK	In-person or telephone assessment by GP physician along with paramedic care, referral to follow-up care in the community or triage to the emergency department if required. Self-care advice, interface/follow-up with intermediate care teams, social services, community hospitals or own registered GP

Citation	Journal or Publication	Study Type	Peer Review	Country	Model of Care Name or Description	Target Population	Geographic and Operational Context	Services Provided
Wagstaff and Mistry, 2020	British Journal of General Practice	Article or Vignette	Yes	Canada	Community paramedic role in GP practices	All people rostered with a primary care practice or organization	Buckingham, UK	Same-day urgent and scheduled home visits to provide routine follow-up care and some acute-on-chronic exacerbations for patients of a primary care practice
Walker et al., 2006	Emergency Medicine Journal	Observational Study	Yes	UK	Diabetes referral pathway following acute hypoglycemic episode	People with diabetes experiencing an acute hypoglycemic episode	Two areas of West Yorkshire, UK	Emergency treatment of hypoglycemia, follow-up with reassessment of diabetes and health education to improve patient's ability to manage diabetes
Washko et al., 2016	EMS World	Article or Vignette	No	USA	Advanced Illness Management Project - community paramedic pilot program	People at high risk for hospital readmission	New York City and Long Island, NY, USA	On-demand clinical care for acute exacerbations of chronic conditions, phone consults, home visit, and emergency transport

Citation	Journal or Publication	Study Type	Peer Review	Country	Model of Care Name or Description	Target Population	Geographic and Operational Context	Services Provided
Watson, 2015	Journal of Emergency Medical Services	Article or Vignette	No	USA	Community Health Access Program - Christian Hospital EMS MIH-CP program; Advanced Practice Paramedics (APPs)	People at high risk for hospital readmission		Home assessment, home treatment, health advice and education, referral to primary care physician or community health centre
Webb et al., 2010	Journal of Forensic and Legal Medicine	Observational Study	Yes	UK	Provision of medical services to detainees in police custody by an external commercial service provider, G4S Forensic & Medical Services	People in police custody	Contract-based provider with contracts with 11 police forces across the UK: Bedfordshire, Cambridgeshire, Devon & Cornwall, City of London, Essex, Gloucestershire, Hampshire, Lincolnshire, Norfolk, Suffolk and Wiltshire	On-scene medical care for full spectrum of conditions as-needed while in police custody; can be escalated to further ED/hospital care if needed
Whalen et al., 2018	Canadian Journal of Emergency Medicine	Qualitative Study	Yes	Canada	Collaborative Emergency Centre (CEC)		Nova Scotia, Canada	Overnight urgent and emergency care within a rural community

Citation	Journal or Publication	Study Type	Peer Review	Country	Model of Care Name or Description	Target Population	Geographic and Operational Context	Services Provided
Widiatmoko et al., 2008	Primary Health Care Research & Development	Observational Study	Yes	UK	Alternate response to low-priority non-urgent ambulance calls, pilot project	People experiencing low-acuity emergencies	"UK setting"	Treatment on scene for non-urgent conditions

Citation	Journal or Publication	Study Type	Peer Review	Country	Model of Care Name or Description	Target Population	Geographic and Operational Context	Services Provided
Wilcox, 2016	EMS World	Article or Vignette	No	USA	Rural community paramedicine	People who are underinsured with high needs, including those with disabilities	Three rural counties in Minnesota, USA: Rice County, Wadena County, and Scott County	Home safety checks, nutritional counseling, medication review, patient assessment and mental health monitoring. Blood draws at home; tracheal tube, stoma care; ostomy care; bladder scans; medication administration to the mentally ill; medication education and review; EKG procurement, IV starts, laceration repair and simple extremity splinting; wound care; home visits to assess for fall hazards; post-surgical visits. Mobile clinic, resource center for food access, job placement and public assistance resources
Willoughby et al., 2005	Hospital Medicine	Article or Vignette	Yes	UK	Integrated cardiac care	People with symptoms of heart attack	Lincolnshire, UK	24/7 emergency treatment for heart attack, transport to specialty team, primary care follow-up after event

Citation	Journal or Publication	Study Type	Peer Review	Country	Model of Care Name or Description	Target Population	Geographic and Operational Context	Services Provided
Young et al., 2014	Healthcare Quarterly	Case Report or Case Study	Yes	Canada	Toronto Heart Attack Collaborative - city-wide integration to enable universal 24/7 access to primary percutaneous coronary intervention	People with symptoms of heart attack	City of Toronto municipal administrative boundary (Etobicoke creek to the west, Steele's ave to the north, Rouge valley to the east, Lake Ontario to the south)	Coordination and quality assurance to receive primary PCI within 90 mins of contact with the health system (including via paramedics) if having a STEMI
Young, 2019	Relias Media	Article or Vignette	No	USA	Focus Hilliard community outreach and case management	People at high risk for hospital readmission	Norwich Township in Hilliard, Ohio, USA	Home visits to assess and meet "various needs", including assessing for fall risk, medication access, or social support
Zayas, 2018	AACN Advanced Critical Care	Article or Vignette	Yes	USA	Critical Care Transport; Mobile Stroke Transport Unit (MSTU)	Cardiac and intensive care patients requiring complex specialist interventions	Cleveland Clinic in Ohio	Treatment and management of stroke or internal brain injury (e.g., hemmorhage), transport and transfer of care from primary facility to specialty centre and subsequent additional treatment
"Community Paramedics Fill Gaps," 2014	Relias Media	Article or Vignette	No	USA		Multiple		

Citation	Journal or Publication	Study Type	Peer Review	Country	Model of Care Name or Description	Target Population	Geographic and Operational Context	Services Provided
"Community Partnership Provides," 2016	Relias Media	Article or Vignette	No	USA	Healthy@Home Community Paramedic Program	Adults with multiple chronic conditions such as CHF, COPD, Diabetes	suburban Atlanta, Georgia, USA	Detailed clinical and psychosocial assessments, health checks and education for patients at home, coordination of community resources, facilitation of needs including transportation, office visits, nutrition, exercise, and lifestyle changes
"ED Staff, Paramedics," 2014	Relias Media	Article or Vignette	No	USA	Advanced Practice Paramedics	High users of the health or 911 system	Wake County, North Carolina, USA	
"Mobile Teams Fill the Gap," 2016	Relias Media	Article or Vignette	No	USA	Mobile Integrated Health	People at high risk for hospital readmission	Ridgewood, New Jersey, USA	Home health assessment, discharge planning and education, medication reconciliation, primary care follow-up, appointment coordination and booking

Citation	Journal or Publication	Study Type	Peer Review	Country	Model of Care Name or Description	Target Population	Geographic and Operational Context	Services Provided
"Mount Sinai Leverages Smartphone," 2015	Relias Media	Article or Vignette	No	USA	Mobile Acute Care Team (MACT)	Patients seeking emergency treatment for specific conditions where treatment can be safely provided at home	Mount Sinai Hospital in New York, NY, USA	Hospital-level inpatient care for select conditions provided at home rather than being admitted to hospital: IV medications, nebulizer treatment, oxygen, reassessment
"Novel Paramedic Programs," 2017	Relias Media	Article or Vignette	No	USA	Community paramedics for mental or behavioural health; Upstream Crisis Intervention	People experiencing mental health emergency	Two separate programs in the USA: Modesto, California; Atlanta, Georgia	On-call for behavioural health crisis, emergency mental health crisis intervention, physical assessment and medical clearance, transition of care to appropriate facility if needed (outpatient follow-up appointment, direct transfer to mental health facility, or ED)
"Trained Paramedics in California," 2016	Mental Health Weekly	Article or Vignette	No	USA	Community paramedicine pilot project, alternate destination behavioural health pilot	People experiencing mental health emergency	Stanislaus County, California, USA	On-scene assessment in case of mental health crisis, triage to mental health facility if eligible
